# Foreign Correspondence

**Published:** 1846-09

**Authors:** 


### THE WESTERN JOURNAL.

New Series.—Vol. VI.—No. III.

LOUISVILLE, SEPTEMBER 1, 1846.

### FOREIGN CORRESPONDENCE.

We would direct the attention of our readers to the second letter, contained in this number, of our foreign correspondent, Dr. David W. Yandell, a graduate of the University of Louisville, and the son of one of our collaborators. We have read it with pleasure, and think that all who turn to it will peruse it with interest. The author is evidently wide awake to the novel medical world, to which he has transferred himself from the backwoods, and is laudably disposed to make his observations available to the friends and fellow-students he has left behind. Our chief object in the present notice, however, is to say that the author will spend a couple of years in the great *emporia* of medical science, in England, Scotland, Ireland, France, and Germany, and that we have engaged him to furnish us regularly with communications for the Journal. Thus we may venture to promise one for every number, and cannot doubt, that they will become more and more interesting, in proportion as his opportunities for observation multiply. In this way we shall be able to put our readers in possession of the earliest European intelligence, including many things which might not reach them through the journals from that country. D.

